# Research on Rainfall Monitoring Based on E-Band Millimeter Wave Link in East China

**DOI:** 10.3390/s21051670

**Published:** 2021-03-01

**Authors:** Siming Zheng, Congzheng Han, Juan Huo, Wenbing Cai, Yinhui Zhang, Peng Li, Gaoyuan Zhang, Baofeng Ji, Jiafeng Zhou

**Affiliations:** 1 Electronics and Communication Engineering Laboratory, Key Laboratory of Middle Atmosphere and Global Environment Observation, Institute of Atmospheric Physics, Chinese Academy of Sciences, Beijing 100029, China; 20191219117@nuist.edu.cn (S.Z.); huojuan@mail.iap.ac.cn (J.H.); zhanggaoyuan407@163.com (G.Z.); fengbaoji@126.com (B.J.); 2 School of Electronics and Information Engineering, Nanjing University of Information Science and Technology, Nanjing 210044, China; peng.li@nuist.edu.cn; 3 College of Earth and Planetary Sciences, University of Chinese Academy of Sciences, Beijing 100049, China; 4 Beijing Institute of Tracking and Telecommunications Technology, Beijing 100094, China; caiwenbinga@126.com (W.C.); zhangyinhui_nudt@163.com (Y.Z.); 5 College of Information Engineering, Henan University of Science and Technology, Luoyang 471023, China; 6 Department of Electrical Engineering and Electronics, University of Liverpool, Liverpool L69 3GJ, UK; Jiafeng.Zhou@liverpool.ac.uk

**Keywords:** electromagnetic wave propagation, E-band millimeter-waves, rain-induced attenuation, rainfall observation

## Abstract

Accurate rainfall observation data with high temporal and spatial resolution are essential for national disaster prevention and mitigation as well as climate response decisions. This paper introduces a field experiment using an E-band millimeter-wave link to obtain rainfall rate information in Nanjing city, which is situated in the east of China. The link is 3 km long and operates at 71 and 81 GHz. We first distinguish between the wet and the dry periods, and then determine the classification threshold for calculating attenuation baseline in real time. We correct the influence of the wet antenna attenuation and finally calculate the rainfall rate through the power law relationship between the rainfall rate and the rain-induced attenuation. The experimental results show that the correlation between the rainfall rate retrieved from the 71 GHz link and the rainfall rate measured by the raindrop spectrometer is up to 0.9. The correlation at 81 GHz is up to 0.91. The mean relative errors are all below 5%. By comparing with the rainfall rate measured by the laser raindrop spectrometer set up at the experimental site, we verified the reliability and accuracy of monitoring rainfall using the E-band millimeter-wave link.

## 1. Introduction

Due to the variety of climates in different regions of China, rainfall has always been a key meteorological element monitored by the meteorological department. The uneven distribution of rainfall over space and time can cause floods and droughts, which have a great impact on human production and life. Therefore, the accurate real-time monitoring of precipitation is essential [1].

Traditional rain gauges do not have high spatial resolution due to in situ measurement [2]. In addition, weather radars are easily affected by ground echoes at low elevation angles, and their measurement results are limited [3]. Studies have shown that millimeter-waves are affected by many factors such as scattering, reflection, and atmospheric absorption in the process of space propagation. Among them, the influence of rainfall is the most obvious. The attenuation of the millimeter-wave signal becomes greater with increasing frequencies [4,5,6]. Based on this feature, meteorological experts have proposed a method of using communication links to monitor near-ground rainfall and retrieve rainfall rate data [7]. This is a complement to traditional rainfall monitoring methods and adds significantly more observation data to the existing network. In addition, the near-ground rainfall monitoring of the microwave link is an advantage compared to the radar and raindrop spectrometer. The microwave link transmitter is an active sensor, and a power law model relating the rainfall rate and attenuation can be adopted for rainfall estimation.

At present, many countries have carried out research on using the rain-induced attenuation characteristics of microwave links to retrieve rainfall, mainly in the frequency range of 15–40 GHz [8,9,10,11]. For example, in Israel, Messer et al. studied the estimation of rainfall rate by commercial microwave links and analyzed various error sources that affect the estimation accuracy, including signal changes caused by antenna wetting and the uncertainty of the attenuation baseline [11,12]. Making use of the existing commercial wireless networks is equivalent to deploying a very high density of weather monitoring sensors and forming wireless environmental sensor networks (WESNs) [13] all over the world. Chwala et al. used attenuation data from commercial microwave links in the high mountains of southern Germany to estimate the near-surface rainfall rates and used spectral time series analysis to detect wet and dry periods [14]. In South Africa, Ahuna et al. evaluated the rainfall rate measurement of 10 locations with a 5 min integration time to obtain its cumulative distribution [15]. These studies have all contributed to the estimation of rainfall rate using microwave links, but they are all based on data collected from low-frequency links. With the rapid development of fifth-generation wireless communication technology (5G), more spectra and wider bandwidth are required. However, the global bandwidth shortage has prompted the exploration of the underutilized millimeter-wave frequency band. Among them, E-band millimeter waves have recently attracted attention, and research on the use of links in this frequency band for rainfall monitoring has gradually increased [16,17,18]. Al-Samman et al. used a 1.8 km 73.5 GHz E-band link to analyze the rainfall rate and rain-induced attenuation in tropical areas [19]. Luini et al. used two 325 m long links in the E-band (73 GHz and 83 GHz) to collect power data, identify rainfall events and eliminate wet antenna effects, as well as provide a higher-precision prediction model [20]. Their experiment also evaluated the accuracy of the statistical prediction model for terrestrial links currently recommended by the ITU-R in predicting rain-induced attenuation along short-distance and high-frequency links in 5G networks.

In general, most of the published experimental studies are based on low-frequency link measurement data, and some are based on E-band link data collected in a few regions [21,22,23,24]. However, the research on rainfall rate retrieval based on practical E-band millimeter-wave links in China is rare. It is necessary to conduct experiments to provide insightful information for the research on the rainfall rate inversion of millimeter wave links in China. We have previously studied the propagation characteristics of low-frequency millimeter waves and the influence of the atmosphere on millimeter wave transmission [5,9,17]. Compared to existing work, the major contributions of this paper are (1) using the data collected by the E-band millimeter wave link built in Nanjing to evaluate the performance of the link to monitor rainfall; (2) overcoming the difficulty that the attenuation baseline is not constant over time by dynamically determining the baseline; and (3) estimating the rain attenuation error caused by the wet antenna effect.

The rest of this paper structure is as follows. Section 2 introduces the system equipment used to build the E-band millimeter-wave link, the auxiliary equipment for collecting rainfall data, and the propagation characteristics of the link. Section 3 introduces the method of processing the experimental data collected by the link, including wet–dry classification and real-time attenuation baseline calculation. Section 4 introduces the estimation method of the wet antenna effect, and finally obtains rain-induced attenuation to retrieve the rainfall rate. Section 5 presents the analysis and discussion of experimental results. Section 6 gives the conclusion.

## 2. Experimental Equipment and Link Propagation Characteristics

**Figure 1:**
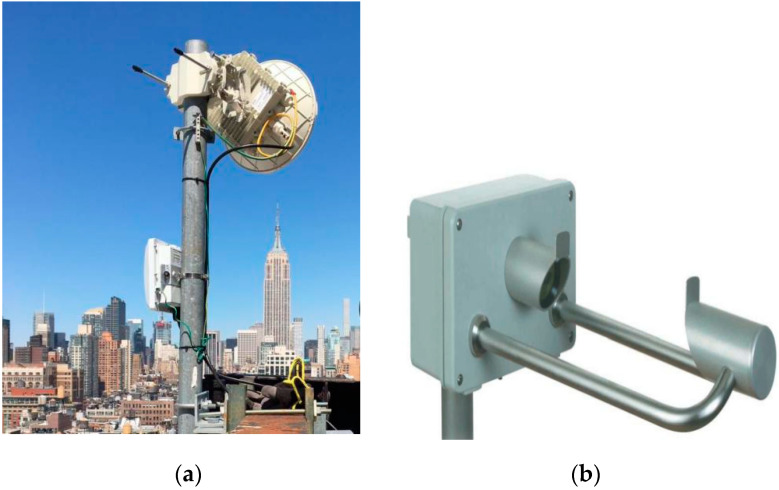
(**a**) E-band radio transceiver; (**b**) the CLIMA laser precipitation monitor.

Figure 1a shows the E-band radio transceiver that we used. This device works in the frequency range of 71–76/81–86 GHz, adaptive modulation, and can directly configure local and remote devices through the network graphical user interface. The details of the microwave link and system operating parameters are described in Table 1.

Figure 1b shows the CLIMA laser precipitation [26] monitor (also called raindrop spectrometer). The time resolution of the data recorded by this instrument is 1 min, which is very suitable for measuring and detecting different types of rainfall, such as drizzle, rainfall, hail, snow and mixed rainfall. The observed particles are divided into 22 diameter categories and 20 velocity categories. We can use this information to calculate the rainfall rate and rain-induced attenuation.

**Figure 2:**
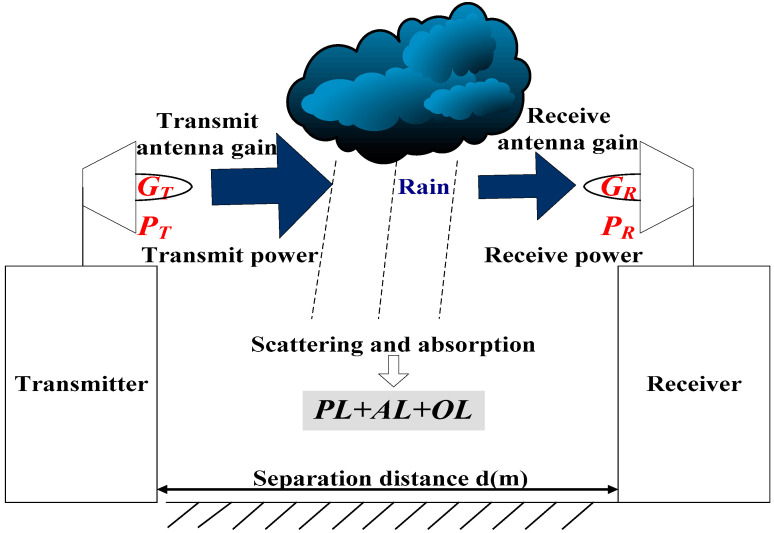
E-band millimeter-wave signal transmission schematic diagram.

On a rainy day, the transmitted wireless signal is attenuated by raindrops due to scattering and absorption, which causes the signal level at the receiver to attenuate, so we can estimate the rainfall rate on the path. Figure 2 shows the schematic diagram of the E-band millimeter-wave signal transmission link composed of a transmitter and a receiver.

The received power $P_{R}$ (dBm) can be expressed as

(1) $$P_{R}=P_{T}+G_{T}+G_{R}-PL-AL-OL$$

where $P_{T}$ (dBm) is the transmitted signal power, $G_{T}$ (dBi), $G_{R}$ (dBi) are the antenna gains of the transmitter and receiver, PL (dB) is the propagation path loss, AL (dB) is the atmospheric loss, and OL (dB) for other losses. PL can be expressed by the following formula [27]:

(2) $$PL\left(f_{c},d\right)=32.4+20\log10\left(f_{c}\right)+10n\log10\left(d/d_{0}\right)+\chi_{\sigma },d\geq 1\;m\;$$

where $f_{c}$ (GHz) is the carrier frequency, d (m) is the distance between the transmitter and the receiver, the reference distance $d_{0}$ is 1 m, and n is the path loss index. $\chi_{\sigma }$ is a zero-mean Gaussian random variable with σ standard deviation, and the unit is dB.

The attenuation model of AL is as follows:

(3) $$AL=A_{r}+A_{v}+A_{o}+A_{p}$$

Atmospheric loss mainly includes the attenuation effects of dry air (including oxygen), water vapor, fog and rainfall. $A_{r}$ (dB) is the attenuation caused by rainfall, $A_{v}$ (dB) is the attenuation caused by water vapor, $A_{o}$ (dB) is the attenuation caused by dry air, and $A_{p}$ (dB) is the attenuation caused by non-rainfall, such as fog, sleet and snow.

Rain-induced attenuation $A_{r}$ and equivalent path-averaged rainfall rate R (mm/h) have a power–law relationship. We can calculate rain-induced attenuation through the simple formula provided in ITU-R P.838-3 [28]. The model is as follows:

(4) $$A_{r}^{ITU-R}=\gamma_{r}^{ITU-R}l=kR^{\alpha }l$$

In the formula, $\gamma_{r}^{ITU-R}$ is the rain-induced attenuation, l is the link length, which is 3 km in this experiment, and k and α are the frequency compliance coefficients, which are related to the millimeter-wave operating frequency, rainfall temperature, polarization mode and raindrop size distribution. In [28], the power–law coefficient corresponding to the 71 GHz link is [k = 1.0409, α = 0.7193], and the power–law coefficient corresponding to the 81 GHz link is [k = 1.1793, α = 0.7004]. Assuming that the rainfall rate is constant along this path, if we obtain rain-induced attenuation, we can also calculate the rainfall rate based on this model. Then, we will introduce the steps and methods for obtaining rain-induced attenuation from the link data.

## 3. Data Processing

### 3.1. Post-Processing

Nanjing has a subtropical monsoon climate with abundant rainfall, which will help us collect more data on rainfall events. Compared with other cities, such as Shanghai and Hangzhou. The humidity in Nanjing area is higher and the wind speed is lower, which has less influence on the antenna, so the experimental equipment is more stable. Therefore, we built an E-band millimeter-wave link in the Nanjing area and collected the received power data from December 2019 to March 2020. The receiver sampled every 1 min with a resolution of 0.1 dB. The link is 3 km long and operates at 71 and 81 GHz. First, the level signal received by the millimeter-wave link is processed. Because the software recording interface of the data acquisition system is frequently updated, if the page is being updated at our sampling time point, the data may not be recorded and be lost. To ensure that the experimental results are not affected, we excluded rainfall events with missing values. We consider 10 rainfall events of different intensity and duration in the filtered experimental data. The dates, duration, total rainfall amounts and maximum rainfall rates (as estimated by the raindrop spectrometer set up at the experimental site) of these events are given in Table 2.

**Figure 3:**
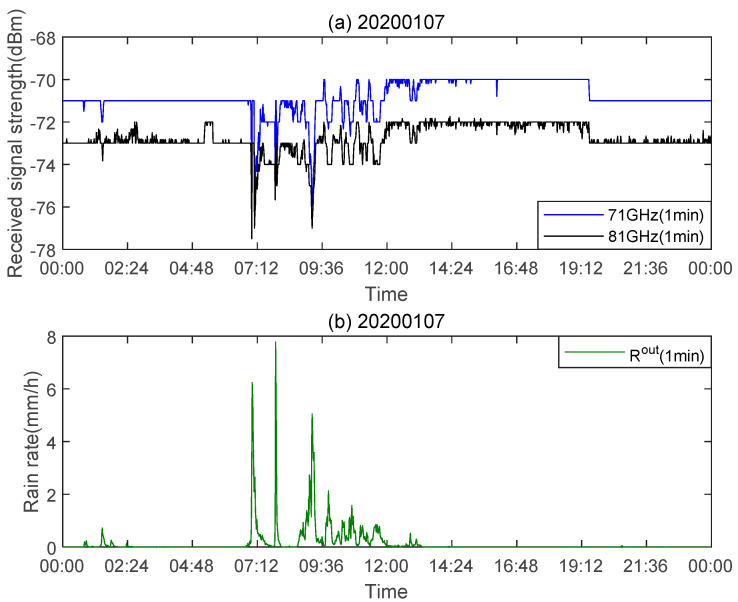
(**a**) The received signal intensity on 7 January 2020; (**b**) the rainfall rate output by the raindrop spectrometer.

The total attenuation value on the link path is obtained by subtracting the transmit power from the received power. This link is a dual-polarization link. The equipment we use can be set to vertical polarization or horizontal polarization. Since the two polarization modes cannot work at the same time, we set the link to be vertically polarized during the experiment. The raindrop spectrometer we use has a time resolution of 1 min for measuring the rainfall rate, which is consistent with the time resolution of path attenuation. Figure 3a shows the signal strength $P_{R}$ received by the E-band link on 7 January 2020. The frequencies are 71 and 81 GHz, respectively. The fluctuation of the receiving level in the dry period in Figure 3a is due to the adaptability of the equipment, and this change is related to its own adjustment [29]. This also greatly increases the difficulty of our data processing. Figure 3b shows the rainfall rate $R^{out}$ output by the raindrop spectrometer set up at the experimental site. It can be seen that after a rainfall event occurs, the received signal intensity is attenuated accordingly, and the rainfall rate is positively correlated with the signal attenuation.

### 3.2. Attenuation Baseline Calculation

The network management system of the equipment we used has an adaptive modulation scheme, which is a transmission mode with better anti-interference and noise immunity, which prevents the link from powering down. When the link is affected by rain, the device will start an adaptive modulation scheme, which causes the reception level before and after rain to not be consistent. From Figure 3a, it can be observed that after the rainfall event ended (approximately at 12:00), $P_{R}$ did not return to the signal strength during the dry period (before the rainfall event), but changed over time after the rainfall event. In the dry period of this day, when the frequency is 71 GHz, $P_{R}$ fluctuates between −72 and −70 dBm, and when the frequency is 81 GHz, $P_{R}$ fluctuates between −74 and −72 dBm. Therefore, the attenuation value during the dry period and the minimum attenuation value cannot be directly used as the baseline. We use the method in [30] to determine the attenuation baseline. Assuming that $A_{T}\left(t\right)$ (dB) is the total attenuation of the link over time, expressed here as

(5) $$A_{T}\left(t\right)=A_{b}\left(t\right)+A_{r}\left(t\right)$$

where $A_{b}\left(t\right)$ (dB) represents the attenuation baseline, and $A_{r}\left(t\right)$ (dB) represents rain-induced attenuation. We define a moving window W=[t−w,t] with a width of w>0:

(6) $${\bar{A}}_{Wt}=\frac{1}{N_{W}}\sum_{k\in Wt}A\left(k\right)$$

(7) $$S_{Wt}^{2}=\frac{1}{N_{W}}{\sum_{k\in Wt}\left(A\left(k\right)-{\bar{A}}_{Wt}\right)}^{2}$$

where $N_{W}$ represents the number of measurements in $W_{t}$. The choice of window size has a great impact on wet and dry classification, and it should not be too large or too small. The moving window between 15 and 30 min can fully represent the dynamics of rainfall [30]. In this range, we compared the link data after wet and dry classification with the raindrop spectrum measurement data. By testing different rainfall events, we found that a 25 min moving window can capture most of the dynamics of rainfall and changes in the attenuation baseline. Therefore, we choose w= 25min. According to the decision rule in [30], for a given threshold $\sigma_{0}$, $S_{Wt}>\sigma_{0}$, it means a rainy period; if $S_{Wt}\leq \sigma_{0}$, it means a dry period.

The value of $\sigma_{0}$ is estimated from attenuation measurements collected during a dry period (usually 24 h). In order to be more robust, we combined several dry periods before the rain, because one dry day is not enough to represent all the variability that affects the link signal during the dry period. Assuming that D represents a dry period and R represents a rainy period, the value of $\sigma_{0}$ is obtained by the following formula:

(8) $$\sigma_{0}=q_{85}\left\{S_{Wt}|t\in D\right\}$$

Among them, $q_{85}$ denotes the 85% quantile, which is the threshold obtained after we analyzed the data of Nanjing from December 2019 to March 2020. Since the rainfall data during our monitoring period accounted for about 15% of all data, we chose a quantile of 85%. In addition, light rain and dry periods show similar variability, so choosing the quantile is more helpful to distinguish them. When the window sizes w and $\sigma_{0}$ are determined, $A_{b}\left(t\right)$ can be determined according to the method in [30]:

(9) $$A_{b}\left(t\right)=\{\begin{array}{c} {\bar{A}}_{Wt},t\in D\\ A_{b}\left(t-m\right),t\in R \end{array}$$

where m is the minimum value that makes t−m∈D. We calculate $\sigma_{0}$ using the dry period before the rainfall event, and the calculated values of ${\bar{A}}_{Wt}$ and $\sigma_{0}$ at the quantile of 85% are shown in Table 3.

## 4. Rainfall Rate Inversion

### 4.1. Raindrop Size Distribution

For rainfall inversion, it is very necessary to find the rainfall rate and related attenuation of the actual rainfall event. We need to know the change of the raindrop size distribution (DSD) in the rainfall of a given intensity. We use the raindrop shape and size function to calculate the rainfall rate $R^{DSD}$ (mm/h) [31] and use $R^{DSD}$ to compare with the inverted rainfall rate in subsequent experiments. According to the DSD data of raindrops, the raindrop density distribution is calculated as follows [32]:

(10) $$N\left(D_{i}\right)=\sum_{j=1}^{20}\frac{N_{ij}}{V_{j}\times S\times T\times \Delta D_{i}}\left[m^{-3}\cdot {\mathrm{mm}}^{-1}\right]$$

where $N_{ij}$ represents the number of raindrops with a diameter at level i and speed at level j, and $D_{i}$ is the diameter of raindrops. S is the sampling area of the raindrop spectrometer, where the value of S is 0.0044 $m^{2}$. T is the sampling time of 60 s, $\Delta D_{i}$ is the diameter interval between two adjacent levels i and (i+1), and $V_{j}$ is the falling speed of the raindrop with a speed of j. The rainfall rate $R^{DSD}$ can be calculated using the formula proposed in [33]:

(11) $$R^{DSD}=6\pi \times 10^{-4}\sum_{i=1}^{22}\sum_{j=1}^{20}V_{j}\times D_{i}^{3}\times N\left(D_{i}\right)\;\Delta D_{i}\left[\mathrm{mm}/h\right]$$

The raindrop shape and size function can be used to not only calculate the rainfall rate, but also express the specific attenuation γ (dB/km) [31]. Mie theory [34,35] is used to calculate the extinction coefficient of a single particle at millimeter-wave frequencies, expressed in integral form as follows:

(12) $$\gamma_{r}^{DSD}=4.343\;\times {\;10}^{3}\;\int_{D}C_{\mathrm{ext}}\left(D,f\right)\;N\left(D\right)\;dD$$

where $C_{\mathrm{ext}}\left(D,f\right)$ ($m^{2}$) is the Mie extinction cross-section with a raindrop diameter of D, which depends on frequency and temperature. It characterizes the scattering and absorption characteristics of each raindrop at a given frequency f and polarization, and determines the attenuation caused by a single raindrop. The application of Mie theory to calculate the extinction cross section also requires a complex refractive index. We use the dielectric function proposed by Liebe et al. [36] which covers the frequency range from 1 to 1000 GHz.

The propagation experiment of Hansryd [37] et al. showed that compared with the low frequency band, the E-band millimeter-wave has a higher scattering efficiency for smaller raindrops and has a stronger dependence on DSD. The model provided in ITU-R P.838-3 shows that there is a power law relationship between the specific attenuation and the rainfall rate, therefore:

(13) $$\gamma_{r}^{DSD}=k^{DSD}R^{DSD}{}^{\alpha^{DSD}}$$

This is a good approximation of the relationship between attenuation and rainfall rate. Our link length l is 3 km, so the rain-induced attenuation $A_{r}^{DSD}$ calculated by DSD can be expressed as

(14) $$A_{r}^{DSD}=\gamma_{r}^{DSD}\times l=k^{DSD}R^{DSD}{}^{\alpha^{DSD}}l$$

**Figure 4:**
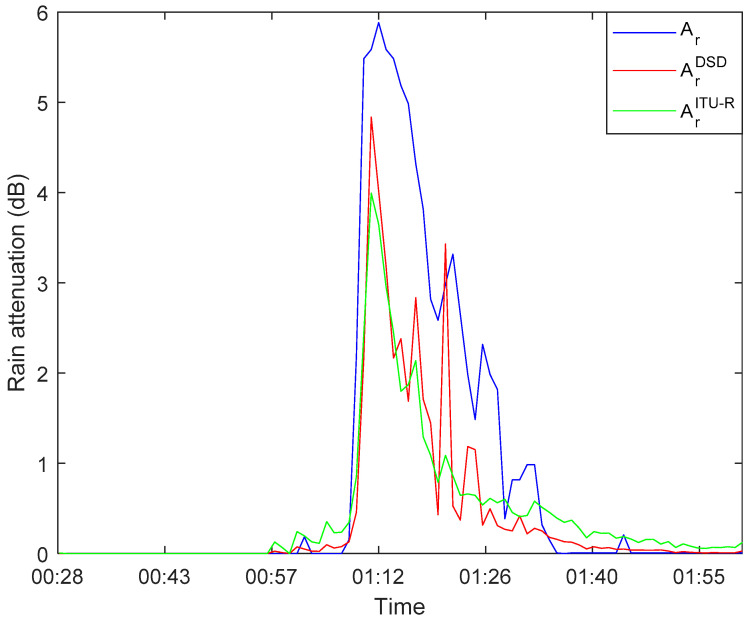
Rain-induced attenuation at 71 GHz on 7 January 2020, blue line: measured link attenuation, red line: estimated from raindrop size distribution (DSD) data, green line: using the method recommended by ITU-R P.838-3.

Figure 4 is the first rainfall event that occurred in the 71 GHz band on 7 January 2020, comparing the three methods outlined above for estimating the rain-induced attenuation. The blue line represents the measured rain-induced attenuation based on the method presented in Section 3, the red line represents the attenuation calculated from the measured DSD data based on Equation (12), and the green line represents the attenuation estimation using the method recommended by ITU-R P.838-3 based on the rainfall rate given by the raindrop spectrometer. It can be seen from Figure 4 that the trends of the three curves are similar. However, the attenuation $A_{r}$ measured by the link, is higher than the other two methods during the rainy period. This deviation directly affects the accuracy of rain-induced attenuation measured by link data. Under normal circumstances, the relative humidity level and temperature level in the environment before and after the rain are similar and will not cause significant attenuation. After excluding the influence of humidity and temperature, the wet antenna effect is the main reason for the difference between the measured rain-induced attenuation and the actual attenuation of the link [38]. We will study methods to eliminate this effect in the following section.

### 4.2. Wet Antenna Correction

When it rains, the water layer adheres to the surface of the reflector, radome and horn cap. In this case, it will cause significant signal attenuation [39,40,41]. Since they are almost vertical surfaces made of hydrophobic materials, the amount of water attached to the surface of the radome may have a maximum value, which will cause the attenuation caused by it to reach a saturated value.

An exponential relationship between the measured attenuation value $A_{r}$ and the attenuation $A_{wa}$ caused by the wet antenna is proposed in [42]. There are two models of dual-frequency model and single-frequency model. The experimental results in [43] show that the single-frequency model we are using is more accurate, as follows:

(15) $$A_{wa}=C\cdot \left(1-\exp\left(-d\cdot A_{r}\right)\right)$$

where C (dB) and d (dB^−1^) are model parameters. Tests have proved that the wet antenna attenuation increases with the lowest value of measured attenuation and rainfall rate, and finally it reaches the saturation value. In this case, C is selected as the representative of the largest difference between the forecast and the measurement observed in the time series. d is calculated by nonlinear regression of the model during the observation period [43].

**Figure 5:**
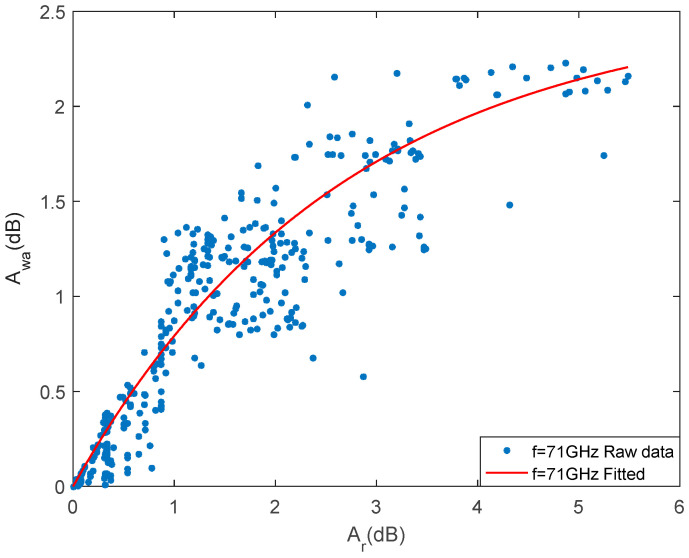
Curve fitting of $A_{r}$ and $A_{wa}$ at 71 GHz.

We used the rainfall event data in January in Table 2 for fitting. It can be seen from Figure 5 that at 71 GHz, when $A_{r}$ is 5.5 dB, the wet antenna attenuation is expected to reach a plateau value. Therefore, the wet antenna attenuation $A_{wa}$ can be expressed as

(16) $$A_{wa}=\{\begin{array}{ll} 2.5283\;(1-e^{-0.3757A_{r}}), & A_{r}\leq 5.5\;\mathrm{dB}\\ 2.25, & A_{r}>5.5\;\mathrm{dB} \end{array}$$

**Figure 6:**
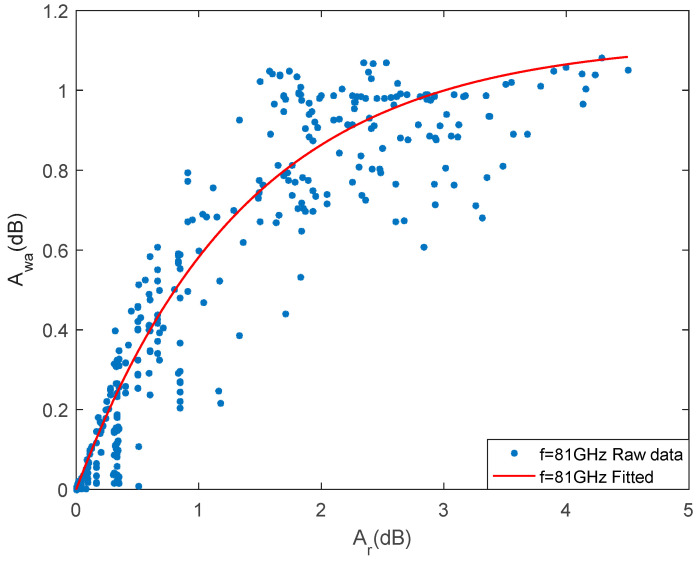
Curve fitting of $A_{r}$ and $A_{wa}$ at 81 GHz.

Similarly, as shown in Figure 6, at 81 GHz, when $A_{r}$ is 4.5 dB, the wet antenna attenuation is expected to reach a plateau value. Therefore, $A_{wa}$ is expressed as follows:

(17) $$A_{wa}=\{\begin{array}{ll} 1.1270\;\left(1-e^{-0.7265A_{r}}\right), & A_{r}\leq 4.5\;\mathrm{dB}\\ 1.1, & A_{r}>4.5\;\mathrm{dB} \end{array}$$

We applied the wet antenna correction model to the link data to predict rain-induced attenuation, and the corrected attenuation level $A_{r}^{\prime }$ can be obtained from $A_{r}^{\prime }=A_{r}-A_{wa}$.

**Figure 7:**
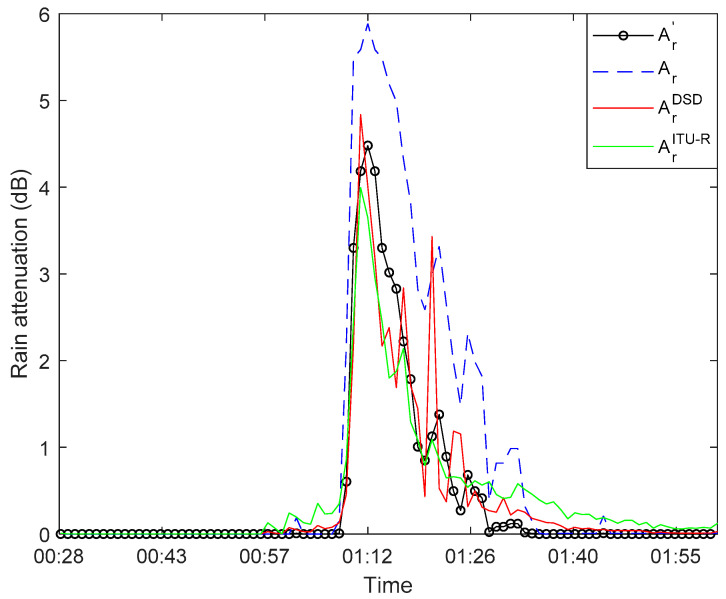
Rain-induced attenuation at 71 GHz on 7 January 2020, black line: rain-induced attenuation after correction; blue line: predicted from link data; red line: estimated from DSD data; green line: using the method recommended by ITU-R P.838-3.

Figure 7 shows the first rainfall event that occurred in the 71 GHz band on 7 January 2020. The rain-induced attenuation after correcting the influence of the wet antenna shows a better fitting effect than before the correction and is closer to the result estimated from the DSD data and estimated using the ITU-R P.838-3 recommendation.

### 4.3. Rainfall Rate Inversion Result

We used the millimeter-wave link to collect data from December 2019 to March 2020 and analyzed the rainfall events in these four months. We then applied the above model to invert the rainfall rate, and evaluated the inversion effect by calculating the Pearson correlation coefficient and the mean relative error. The formula is as follows:

(18) $$r_{k}\left(X_{i},Y_{i,k}\right)=\frac{1}{N-1}\sum_{i=1}^{N}\left(\frac{X_{i}-\mu_{X}}{\sigma_{X}}\right)\left(\frac{Y_{i,k}-\mu_{Y}}{\sigma_{Y}}\right)$$

(19) $$MRE_{k}=\frac{100\%}{N}\times \sum_{i=1}^{N}\left|\frac{X_{i}-Y_{i,k}}{X_{i}}\right|$$

**Figure 8:**
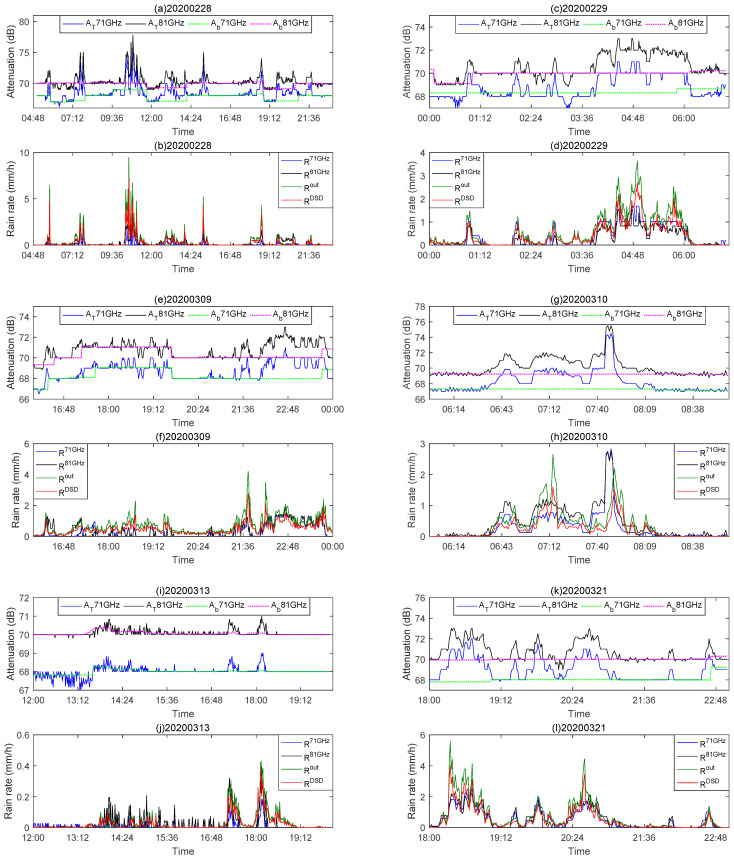
The total attenuation and baseline of 71 and 81 GHz on different days. The comparison result of the link retrieved the rainfall rate $R^{71\;\mathrm{GHz}}$ and $R^{81\;\mathrm{GHz}}$, raindrop spectrometer output rainfall rate $R^{out}$ and DSD data to calculate rainfall rate $R^{DSD}$.

Among them, $X_{i}$ represents the rainfall rate $R^{M}$ estimated by the link data. When *k* is 1, $Y_{i,1}$ represents the rainfall rate $R^{out}$ the output by the raindrop spectrometer, and when *k* is 2, $Y_{i,2}$ represents the rainfall rate $R^{DSD}$ calculated by the DSD. $\mu_{X}$ and $\sigma_{X}$ are the mean and standard deviation of $X_{i}$, respectively, and $\mu_{Y}$ and $\sigma_{Y}$ are the mean and standard deviation of $Y_{i,k}$, respectively. A higher correlation coefficient and lower mean relative error is desired. This means that there is better similarity between the two data sets, indicating that the rainfall rate estimation from the millimeter-wave link can represent the true rainfall rate well. Figure 8 shows the total attenuation and baseline and rainfall rate results. $A_{T}71\;\mathrm{GHz}$ and $A_{T}81\;\mathrm{GHz}$ represent the total attenuation of 71 and 81 GHz received signals, and $A_{b}71\;\mathrm{GHz}$ and $A_{b}81\;\mathrm{GHz}$ represent the attenuation baseline. Table 4 lists the 6-day rainfall rate correlation coefficient and mean relative error value.

## 5. Discussion

From the experimental results, the accuracy of the rainfall rate retrieved from millimeter-wave link data is relatively high. As shown in Table 4, the mean relative errors are all below 5%, and the correlations for five days in the 81 GHz link are above 0.6, which shows that the millimeter-wave link in the E-band can monitor rainfall well. Figure 8 shows the received signal strength and rainfall rate of the day. It can be seen that as the rainfall event occurs, the signal strength is attenuated accordingly. The $R^{71\;\mathrm{GHz}}$ and $R^{81\;\mathrm{GHz}}$ shown in Figure 8 are the results of eliminating the wet antenna effect. The retrieved rainfall rate is lower than the raindrop spectrometer output rainfall rate $R^{out}$ and the DSD data to calculate rainfall rate $R^{DSD}$ when the rainfall is heavy. The determination of the attenuation baseline allows the influence of humidity and temperature to be excluded, so this may be related to the excessive elimination of attenuation when removing the effect of the wet antenna, which means that part of the attenuation caused by the wet antenna will be eliminated when the method in Section 3.2 correctly extracts the rain-induced attenuation. The same conclusion is mentioned in [20].

The link adaptive feature of the wireless transceiver can increase the transmission power and can also use a most robust modulation scheme to maintain the link when the channel quality is poor. As shown in Figure 8e, this feature may have caused the rain-induced attenuation to be lower than its level during dry period. As discussed in [44], the millimeter-wave link is designed for effective communication services and not for monitoring rainfall. Therefore, link instability increases the difficulty for our research. From Table 4, we can also see that the mean relative error of $R^{71\;\mathrm{GHz}}$, $R^{81\;\mathrm{GHz}}$ and $R^{DSD}$ is about 0.01–1.48 % lower than the mean relative error of $R^{out}$, and this difference is relatively small. This shows that the difference between $R^{out}$ and $R^{DSD}$ will not have a great impact on the results of rainfall retrieval. However, in order to retrieve rainfall more accurately, this cannot be easily ignored.

**Figure 9:**
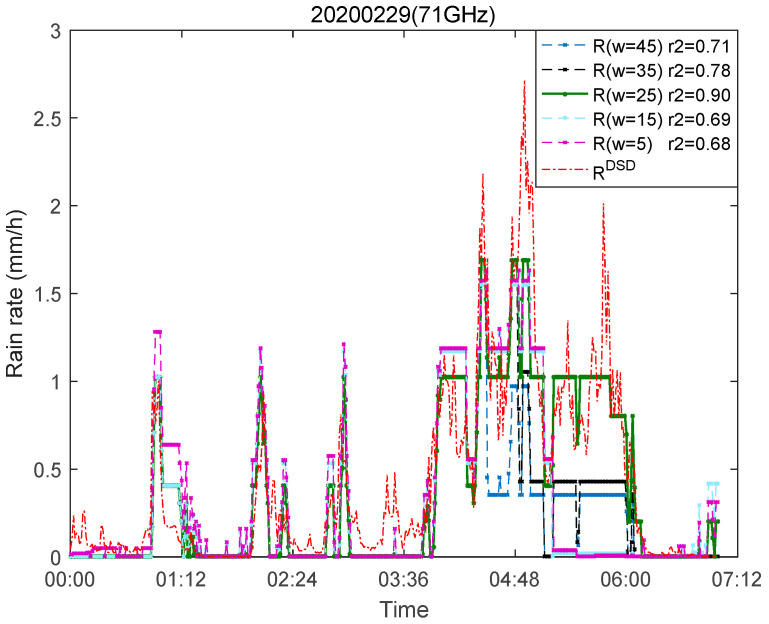
Rainfall rate is retrieved for links in different time windows at 71 GHz on 29 February 2020, and $r_{2}$ represents the correlation between R (71 GHz) and $R^{DSD}$.

Taking the rainfall event on 29 February 2020 as an example, it can be seen from the data of $R^{out}$ and $R^{DSD}$ in Figure 8d that there is some drizzle since 00:00 on this day. We found that for small rainfall, the received signal strength of millimeter-wave has small fluctuations, which can be seen from Figure 8c. However, this is a fluctuation lower than the attenuation baseline, and the retrieved rainfall rate is not reflected. From the 81 GHz signal receiving strength in Figure 8c, it can be seen that the small fluctuations from 00:00 to 01:00 are higher than the attenuation baseline, which is also reflected in the rainfall retrieval results. This may be related to the size of the time window selected in Section 3.2, which affects the determination of the attenuation baseline. As shown in Figure 9, if we choose a small window, this attenuation will be reflected. However, the disadvantage is that it will overestimate the rain-induced attenuation, thereby reducing the accuracy of rainfall retrieval result. The correlation $r_{2}$ in Figure 9 is highest when w=25 indicates that the window size we choose is appropriate, which also shows that this rainfall retrieval model is accurate and effective.

## 6. Conclusions

Microwave backhaul links in commercial communication networks are installed and operated all over the world. They can be treated as millions of virtual weather sensors for rainfall monitoring with no extra costs for installation and maintenance. This network based on microwave backhaul links can provide supplementary information on important environmental variables in areas with low traditional monitoring network density, especially in developing countries. This article introduces the research results of the E-band millimeter-wave link built in Nanjing, located in Eastern China. Signals at E-band experiences greater attenuation by rainfall. We use a 3 km-long link to collect measurement data with high time resolution within 1 min sampling interval. Therefore, the link’s ability to accurately quantify the rainfall rate during light rainfall is stronger than that of the traditional microwave links operating at 15–40 GHz. The measurement data are collected with a high time resolution at a 1 min sampling interval.

The experimental results show that the method of separating rain-induced attenuation and eliminating wet antenna attenuation studied in the low frequency band is also applicable to the E frequency band. The mean relative errors are all below 5%, and the correlations for five days in the 81 GHz link are above 0.6. The correlation between the retrieved rainfall rate and the rainfall rate measured by the raindrop spectrometer at 71 GHz is up to 0.9. The correlation at 81 GHz is up to 0.91. This further confirms the high sensitivity of the E-band millimeter-wave link to light rainfall. The determination of the attenuation baseline is very important for accurately separating the rain-induced attenuation. This article uses a simple wet and dry classification and then determines the baseline method. Compared with the model provided by ITU-R P.838-3, the rain-induced attenuation estimated by this method is closer to the calculation result of DSD data, indicating that this method is accurate and effective. In our research, the attenuation caused by the wet antenna is estimated through DSD data and an effective model. The attenuation caused by the wet antenna in a rainfall event is about 2 dB, which is a relatively small value, indicating that the impact of the wet antenna attenuation on the rainfall rate retrieval in the E-band is less than the low frequency band of 15–40 GHz. For a long E-band millimeter-wave link, since we cannot always assume that the rainfall rate along its path is uniformly distributed, this may cause some errors, which is also a factor that affects the experimental results. In a sufficiently long link, it is also possible to separate the attenuation caused by water vapor, which of course is challenging in practice. Since we are currently mainly studying rainfall inversion, there is no solid precipitation in the rainfall event we are considering, so we have not classified the precipitation type. In future research, we plan to build two other links for precipitation classification or consider using machine learning methods to classify precipitation for a single link.

## Figures and Tables

**Table 1:** System parameters of the E-band link [25].

Parameter	71–76/81–86 GHz
Transmit power	+7 dBm
Level resolution	0.1 dB
Tx and Rx antenna gain	50 dBi
Antenna polarization	vertical
Antenna size	0.65 m
Bandwidth	250 MHz
Throughput	1 Gbps full duplex
Link budget (BER (Bit Error Rate) = 10^−6^)	196 dB (including 2ft antennas’ gain)
Modulation	QPSK (Quadrature Phase Shift Keying)

**Table 2:** Date, duration (in hours), rain amount (in millimeters), and maximum rainfall rate (in millimeter per hour) of the considered rain events.

Date	Duration (h)	Accumulated Rainfall Rates (mm/h)	Maximum Rainfall Rates (mm/h)
7 January 2020	6.5	196.7	7.7
22 January 2020	4.5	274	3.6
24 January 2020	5.5	20	0.4
25 January 2020	9	154	1
28 February 2020	17	407	9.4
29 February 2020	7	248	3.6
9 March 2020	8	337	4.2
10 March 2020	3	64	2.7
13 March 2020	7	13	0.4
21 March 2020	5	220	5.6

**Table 3:** Calculated values of ${\bar{A}}_{Wt}$ and $\sigma_{0}$ obtained from the data of the considered dry periods.

Date (Dry)	Frequency	${\bar{A}}_{Wt}\left(q\;=\;85\%\right)$	$\sigma_{0}\left(q\;=\;85\%\right)$
31 December 2019	71 GHz	69.2	0.03
	81 GHz	70.5	0.05
19 January 2020	71 GHz	67.8	0.03
	81 GHz	69.9	0.04
24 February 2020	71 GHz	68.6	0.08
	81 GHz	70.1	0.08
7 March 2020	71 GHz	68.4	0.09
	81 GHz	70.4	0.09
11 March 2020	71 GHz	67.9	0.05
	81 GHz	69.9	0.06
18 March 2020	71 GHz	68.5	0.08
	81 GHz	69.9	0.08

**Table 4:** Correlation ($r_{1}$ and $r_{2}$) and mean relative error ($MRE_{1}$ and $MRE_{2}$) of the estimated rainfall rate (based on the 71 and 81 GHz link) and the rainfall rate recorded by raindrop spectrometer.

Date	Frequency	$R^{\mathrm{out}}$		$R^{\mathrm{DSD}}$	
		$r_{1}$ (-)	$MRE_{1}$ (%)	$r_{2}$ (-)	$MRE_{2}$ (%)
28 February 2020	71 GHz	0.90	4.02	0.89	3.44
	81 GHz	0.91	0.99	0.89	0.68
29 February 2020	71 GHz	0.88	0.57	0.86	0.40
	81 GHz	0.89	3.69	0.87	2.21
9 March 2020	71 GHz	0.59	2.94	0.61	1.95
	81 GHz	0.57	0.78	0.60	0.53
10 March 2020	71 GHz	0.55	0.35	0.53	0.26
	81 GHz	0.67	0.24	0.65	0.23
13 March 2020	71 GHz	0.59	0.58	0.61	0.47
	81 GHz	0.63	0.48	0.63	0.40
21 March 2020	71 GHz	0.87	0.52	0.87	0.36
	81 GHz	0.84	3.14	0.84	2.22

## Data Availability

The data presented in this study are available on request from the corresponding author. The data are not publicly available due to restrictions privacy.
